# Navigation guidance in neuroendoscopic management of complex hydrocephalus

**DOI:** 10.3171/2023.1.FOCVID22152

**Published:** 2023-04-01

**Authors:** Nishanth Sadashiva, Subhas Konar, Chirag Jain, Dhaval Shukla

**Affiliations:** Department of Neurosurgery, National Institute of Mental Health and Neurosciences (NIMHANS), Bengaluru, India

**Keywords:** electromagnetic navigation, multiloculated hydrocephalus, neuroendoscopy

## Abstract

Patients with multiloculated hydrocephalus have multiple, separate abnormal CSF collections with no communication between them. Causes include complications of neonatal meningitis, germinal matrix hemorrhage in neonates, head trauma, and intracranial surgery. Endoscopic fenestration with shunt insertion is a safe and less invasive technique as the initial treatment. In this video, the authors demonstrate a few cases in which electromagnetic navigation was used with a stylet inserted through the operating endoscope to guide the surgeon. Modalities such as insertion of intraventricular contrast and fluorescein may be used as adjuvants, as demonstrated. The use of navigation helps to identify distorted anatomical landmarks and guides surgery.

The video can be found here: https://stream.cadmore.media/r10.3171/2023.1.FOCVID22152

## Transcript

In this video, we demonstrate navigation guidance in neuroendoscopic management of complex hydrocephalus. Patients with multiloculated hydrocephalus have multiple, separate CSF collections. Causes include complications of neonatal meningitis, germinal matrix hemorrhage, head trauma, and intracranial surgeries. Options include insertion of multiple shunts or fenestration of septa either by open craniotomy or by endoscopy. Endoscopic fenestration with shunt is a safe and less invasive technique. Distortion of normal anatomy and choosing different entry sites leads to difficulty navigating inside the cysts. Majority of these patients are young, and hence electromagnetic navigation is used frequently. Continuous intraoperative irrigation leads to less collapse of the ventricle and therefore less brain shift.^1–5^

### 1:13 Case 1.

A 10-month-old female child was operated for ruptured meningomyelocele at birth. Subsequently, patient underwent two ventriculoperitoneal shunts, which were infected, and both shunts were removed due to meningitis. MRI showed multiseptate hydrocephalus. Patient was positioned supine, head resting on a soft jelly ring, and electromagnetic neuronavigation registration was done. Endoscope was introduced after making a burr hole in the right temporal region. Electromagnetic navigation stylet is introduced through the operating scope to navigate inside the cyst. Endoscope is navigated inside the cyst with the use of navigation system. Once inside, we moved to check the optimum site and trajectory where subsequent cysts can be punctured and communicated. The membrane is coagulated. After coagulation, the aperture is enlarged using Fogarty balloon catheter inflation. After an adequate aperture is done, the endoscope is introduced through the hole to communicate to another cyst. Here we navigate in another cyst so that we can identify subsequent cysts which are located more superiorly, so that those can be perforated as well. The membranes separating the subsequent cysts are coagulated fenestrations enlarged for proper communication of these cysts. In some areas, we may encounter multiple membranes in multiple layers, which needs to be patiently perforated and communicated. Navigation is used to confirm proper entry to the superior cyst. Subsequently, we move inferiorly through the temporal cyst to reach the area around the basal cisterns. The adhesions and thin membranes are coagulated and divided. As evident here, these septations can be found in multiple layers and navigation will help in identifying the depth as well as direction in which dissection may have to be done. It is also important to choose avascular areas of septa to avoid bleeding and to keep the media clean. Entry into the prepontine space has to be confirmed using navigation system. Finally, a ventriculoperitoneal shunt catheter is introduced through the endoscope passing through multiple cysts. A CT after surgery showed optimum positioning of VP shunt catheter.

### 4:33 Case 2.

A 9-month-old child with neonatal meningitis had multiloculated hydrocephalus for which multiple shunt revisions were done. Currently, a left-side Frazier’s point shunt was in situ draining the cyst, but right temporal horn was trapped with few cysts. A CT showed loculated right temporal horn and decompressed ventricle system due to a shunt in the left-side ventricles. Patient underwent navigation-guided endoscopic surgery. Endoscope was introduced into the trapped right temporal horn through a burr hole. Then through an aperture a cystic membrane was identified. The membrane was coagulated, fenestrated, and enlarged by a Fogarty balloon catheter. Subsequently, deeper cyst wall was identified and membrane fenestrated in an optimum trajectory. Some of the thicker membranes may cause bleeding during fenestration, which can be easily controlled by either coagulation or insufflating fluid. The entry into deeper loculate was confirmed using navigation system. Finally, radiopaque contrast material was injected through endoscope for imaging confirmation. A CT showed contrast material in the intended opposite side, confirming communication of the cyst.

### 6:15 Case 3.

In the next case, we present a 1-year-old child with postmeningitic multiloculated hydrocephalus. MRI showed multiloculated hydrocephalus with a large left occipital cyst. In the immediate preoperative period, right frontal horn was tapped and fluorescein was injected to aid in intraoperative identification. Left occipital horn was entered using an endoscope, and the endoscope was navigated inside the ventricles to identify sites of other cysts. After identifying the site of temporal cyst using navigation, the membrane was perforated and communicated with the rest of the ventricle system. In this case, the navigation stylet itself was used for perforating the membrane. After perforating, enlargement of perforation was done using Fogarty balloon catheter. Subsequently, the endoscope is directed toward the frontal horn where fluorescein could be identified beyond the membrane. This membrane was fenestrated and communication confirmed by spillage of fluorescein dye across the membrane. Finally, a VP shunt catheter was introduced into the frontal horn to convert into ventriculoperitoneal shunt system.

### 7:53 Case 4.

In the next case, we present a 10-year-old child with congenital hydrocephalus with eight previous VP shunt revisions since infancy including ventriculopleural shunt, resulting in a pleural effusion and lung collapse which required surgery. Previous history of ventriculitis was present, which was treated with antibiotics. Repeat shunting was not an option due to abdominal adhesions and hydrothorax. MRI showed hydrocephalus with aqueductal obstruction, and the floor of third ventricle appeared very thick. Right frontal burr hole was done and ventricles entered. The floor of third ventricle appeared thick and anatomy was distorted. Navigation system was used to identify the exact expected entry point for third ventriculostomy. The floor was perforated, the deeper area inspected and confirmed with navigation. Navigation had to be used again to check the entry into prepontine space. The membrane appeared very thick, so the perforation was enlarged using Fogarty balloon to prevent any reocclusion. Flapping of the floor of third ventricle was seen. Postop, patient improved and has been shunt independent for more than a year.

### 9:06 Follow-Up.

This an MRI of a typical patient with multiloculated hydrocephalus who underwent endoscopic fenestration of cysts and ventriculoperitoneal shunt. Over time, there is decrease in cyst size and expansion of brain matter.

### 9:19 Conclusions.

In conclusion, neuronavigation guidance is feasible for neuroendoscopic management in cases with complex hydrocephalus. Modalities like intraventricular contrast as well as fluorescein may be added as adjuvants, as demonstrated, for confirmation of connections between loculations. The use of navigation helps to identify distorted anatomical landmarks and guides surgical procedure.

## Disclosures

The authors report no conflict of interest concerning the materials or methods used in this study or the findings specified in this publication.

## Author Contributions

Primary surgeon: Sadashiva, Konar, Shukla. Assistant surgeon: Jain. Editing and drafting the video and abstract: Sadashiva, Konar, Jain. Critically revising the work: Sadashiva, Konar, Shukla. Reviewed submitted version of the work: Sadashiva, Konar, Shukla. Approved the final version of the work on behalf of all authors: Sadashiva. Supervision: Shukla.

## Supplemental Information

### Patient Informed Consent

The necessary patient informed consent was obtained in this study.
